# Elemental imaging (LA-ICP-MS) of zebrafish embryos to study the toxicokinetics of the acetylcholinesterase inhibitor naled

**DOI:** 10.1007/s00216-018-1471-2

**Published:** 2018-11-16

**Authors:** Katharina Halbach, Stephan Wagner, Stefan Scholz, Till Luckenbach, Thorsten Reemtsma

**Affiliations:** 1 Department of Analytical Chemistry, Helmholtz Centre for Environmental Research - UFZ, Permoserstraße 15, 04318 Leipzig, Germany; 2 Department of Bioanalytical Ecotoxicology, Helmholtz Centre for Environmental Research - UFZ, Permoserstraße 15, 04318 Leipzig, Germany; 3 Institute of Analytical Chemistry, University of Leipzig, Johannisallee 29, 04103 Leipzig, Germany

**Keywords:** Elemental distribution, Enzyme inhibition, Quantitative bioimaging, Biotransformation, Bioaccumulation, Reactive toxicity

## Abstract

The zebrafish embryo is an important model in ecotoxicology but the spatial distribution of chemicals and the relation to observed effects is not well understood. Quantitative imaging can help to gain insights into the distribution of chemicals in the zebrafish embryo. Laser ablation inductively coupled plasma-mass spectrometry (LA-ICP-MS) is used to quantify the uptake and the uptake kinetics of the bromine (Br) containing organophosphate naled (Dibrom®, dimethyl-1,2-dibromo-2,2-dichloroethylphosphate) and its distribution in zebrafish embryos using Br as the marker element. During exposure, the Br amounts increase in the embryos parallel to the irreversible inhibition of the acetylcholinesterase (AChE). The final amount of Br in the embryo (545 pmol/embryo) corresponds to a 280-fold enrichment of naled from the exposure solution. However, LC-MS/MS analyses showed that the internal concentration of naled remained below the LOD (7.8 fmol/embryo); also the concentration of its known transformation product dichlorvos remained low (0.85 to 2.8 pmol/embryo). These findings indicate the high reactivity and high transformation rate of naled to other products than dichlorvos. ^12^C normalized intensity distributions of Br in the zebrafish embryo showed an enrichment of Br in its head region. Kernel density estimates of the LA-ICP-MS data were calculated and outline the high reproducibility between replicated and the shift in the Br distribution during exposure. The Br enrichment indicates a preferential debromination or direct covalent reaction of naled with AChE in this region.

**Electronic supplementary material:**

The online version of this article (10.1007/s00216-018-1471-2) contains supplementary material, which is available to authorized users.

**Graphical abstract:**
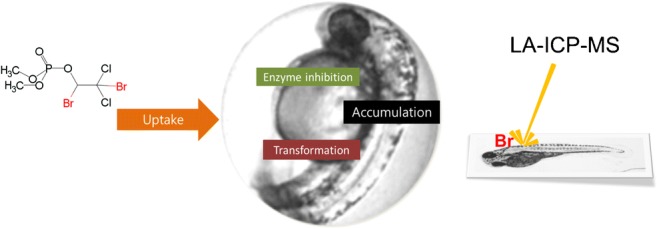
ᅟ

## Introduction

Zebrafish (*Danio rerio*) early life stages (0–ca. 120 h post fertilization (hpf)) represent an attractive small scale in vivo experimental and alternative model to adult animals [1] used in various research fields, such as pharmacology, (eco)toxicology, and genetics [2–4]. Toxicokinetic studies are of essential importance for the understanding of how the external exposure concentration of a toxicant translates into the internal concentration and, possibly, into an adverse outcome [5]. For example, the uptake of some compounds into the tissue depends on the pH of the exposure solution resulting in a seemingly pH-dependent toxicity of these compounds [6]. Furthermore, for compounds with a specific toxic action the effect relates to the concentration at the target site, which is either the receptor with which the toxicant interacts or the reactive site to which it binds, rather than to the average internal concentration of the organism/organ.

Total internal concentrations in zebrafish embryos are typically determined from whole body homogenates, by extraction of the analyzed chemical and subsequent analysis with inductively coupled plasma-mass spectrometry (ICP-MS), liquid chromatography- (LC) or gas chromatography-MS [7–12]. However, the small size of the zebrafish embryo hampers the analysis of a chemical’s spatial distribution given that it is not possible or difficult to dissect organs or tissues. The spatial distribution of chemicals in zebrafish embryos may, instead, be studied by quantitative imaging of molecular or elemental constituents. Chemical imaging could substantially contribute to a better understanding of toxicokinetics in zebrafish embryos, as it would show how chemicals distribute in the zebrafish embryo and whether or not they enrich in specific regions/organs. Such information would be essential to associate biological effects of chemicals with the local concentration at their site of effect.

Mass spectrometry imaging for localizing chemicals in the zebrafish embryo has been performed only recently in a few studies with lipids and exogenous compounds (e.g., cocaine, copper) and nanoparticles [10, 13–15]. The most reproducible quantitative results in MS bioimaging can be obtained with laser ablation (LA) ICP-MS [16] which was applied in the past years to study distributions of natural elements [17–21] or elemental contaminants [22] in biological matrices and to quantify local chemical concentrations [17, 23–25]. The technique determines the elemental composition of a biological sample; it thus can be applied in analyses using elemental constituents as markers, e.g., for the presence of certain proteins, for disease diagnosis, for the mode and potential of biotransformation of a compound, and/or for occurrence of reactive toxicity [26, 27]. The quantification of elements is usually performed either with matrix-matched calibration [28] or with matrix-similar substances such as gelatin [29] or agarose [13, 30].

For obtaining unambiguous results when imaging a biological sample with mass spectrometry, such as LA-ICP-MS, it is crucial that (i) the spatial resolution of the method is adequate to provide a sufficiently detailed image of the object under study. Thus, requirements in spatial resolution increase with decreasing size of this object, e.g., from a fish to a fish embryo; (ii) the specificity of the method is sufficient to detect the chemical in the complex biological matrix; (iii) the sensitivity is sufficient for tissue concentrations that can be low; (iv) quantification is possible, also taking into account matrix effects from the biological sample; (v) internal standardization of the imaging data is possible to account for varying tissue density and composition and for instrumental instability; and (vi) for reactive toxicants, both the primary chemical and the transformation product formed by the reaction with the biogenic target can be detected.

This study shows how quantitative LA-ICP-MS imaging combined with complementary techniques such as LC-MS/MS, nebulization- (neb) ICP-MS and ion chromatography (IC) can be used to study the toxicokinetics of a reactive toxicant in the zebrafish embryo. The study was performed with naled (1,2-dibromo-2,2-dichloroethyl) dimethyl phosphate, Dibrom®), as an organophosphate model insecticide containing the heteroatom bromine (Br) and thus enabling the detection of the compound by ICP-MS. The analytical results on tissue concentrations and localization were combined with the determination of its biological effect, the inhibition of the acetylcholinesterase (AChE). We aimed to (i) quantify the exposure time-dependent naled tissue concentration in relation to its AChE inhibition and transformation, (ii) determine the site (tissue/organ) of transformation, and (iii) statistically compare the LA-ICP-MS results for different individuals exposed to naled for different durations by calculating Kernel densities of the frequencies of pixel intensities. We also tested the hypothesis that Br serves as a marker for naled, i.e., whether the analysis of Br may inform about naled tissue levels.

## Material and methods

### Culture of zebrafish, collection of eggs, and culture of embryos

Zebrafish (*Danio rerio*, Hamilton-Buchanan, 1822), descendents from fish obtained from a local hardware store (generation F13 and F14 from the strain “UFZ-OBI”) were cultured and used for the production of embryos as described previously [31]. Husbandry and experimental procedures were performed in accordance with the German animal protection standards and were approved by the Government of Saxony, Landesdirektion Leipzig, Germany (DD24-5131/25/7).

### Exposure experiments

Naled ((1,2-dibromo-2,2-dichloroethyl) dimethyl phosphate, CAS no. 300-76-5) was dissolved in dimethyl sulfoxide (DMSO) and then diluted with ISO standard dilution water [32] (ISO-water) to the intended exposure concentration (DMSO concentration was 0.02% (*v*/*v*) in all treatments). Concentrations were selected to determine the effect concentrations for AChE inhibition. Based on the results (see the Electronic Supplementary Material (ESM) Fig. S1), a concentration of 3.7 μmol/L naled (corresponding to an AChE inhibition of ca. 45% and approximately 25% of the LC50 [33]) was chosen for subsequent time-resolved effect and spatial analysis. Exposure times of zebrafish embryos were varied from 0.5 to 24 h, with the termination of exposure at 96 hpf to enable measurements at the same developmental stage: 72–96, 86–96, 92–96, 94–96, 95–96, and 95.5–96 hpf. Exposure experiments were performed with 50 zebrafish embryos in a volume of 100 mL exposure solution in three replicates; three negative control experiments were conducted by placing 50 zebrafish embryos in 100 mL ISO-water, respectively. Depuration of naled from the embryo was analyzed by transferring the embryo to control medium [32] after an exposure of 24 h and measurement of naled concentration after 1 and 24 h incubation.

### Measurements of the AChE activity

The AChE enzyme activity in zebrafish embryos tissue extracts was colorimetrically determined [34]. The total amount of protein in tissue extracts was quantified according to Lowry et al. [35]. Twenty zebrafish embryos were placed in FastPrep tubes, washed with 1 mL Milli-Q water, flash frozen in liquid N_2_ and stored at − 20 °C until analysis. Prior to the measurement, 400 μL cold phosphate-buffered saline (PBS, pH 7.7, 0.1 M containing 0.1% *v*/*v* Triton X-100) were added to the embryos, and the embryos were homogenized in a FastPrep®-24 (MP Biomedicals, 6 UxS^−1^, 30 s) and centrifuged at 4 °C for 15 min (13,200 rpm). The supernatant was taken for the enzyme assay and the protein determination. For the enzyme assay, 50 μL of the sample, 50 μL PBS buffer, 100 μL 5,5′-dithiobis-(2-nitrobenzoic acid), and 100 μL acetylthiocholine iodide were added onto a 96-well plate. The absorbance was measured at 412 nm, 22 °C for 10 min. For the protein assay (Bio-Rad *DC*), 5 μL buffer, standard (bovine serum albumin in PBS buffer) or sample was pipetted into a 96-well plate, 25 μL reagent A’ (0.02% *v*/*v* reagent surfactant solution in alkaline copper tartrate solution) and 200 μL Folin reagent were added. After 20 min, the absorbance was measured at 750 nm. In the results, the specific AChE activity was normalized to the mean of the negative controls.

### Sample preparation for LA-ICP-MS and internal concentration analysis

For LA-ICP-MS, zebrafish embryos were washed once with 1 mL Milli-Q water, placed on glass slides and dried at room temperature (RT) for a minimum of 72 h up to a maximum of 144 h. Dried embryos were up to 150 μm in height.

For determining the internal concentration of naled and dichlorvos, 18 zebrafish embryos were placed in FastPrep tubes with glass beads, washed with Milli-Q water, and then frozen in liquid N_2_. For extraction of the chemicals from the embryos, 540 μL methanol (MeOH) were added to each tube. Zebrafish embryos were homogenized in a FastPrep®-24 (MP Biomedicals, 6 m/s, 20 s), placed in an ultrasonic bath (15 min, RT) and centrifuged (13,000 rpm, 15 min, RT). The supernatant was placed in HPLC glass vials and stored at − 20 °C until analysis with LC-MS/MS. For IC measurements of bromide, 200 μL of the supernatant was transferred into a vial and placed for 2 h under a N_2_ gas stream to evaporate the solvent. Afterwards, 200 μL of Milli-Q water was added. For the independent analysis of total bromine with Neb-ICP-MS, 200 μL of the supernatant was taken and diluted with 4 mL of Milli-Q water.

### Instrumentation

#### LA-ICP-MS

LA-ICP-MS measurements were conducted with an Analyte G2 (Teledyne CETAC Technologies Inc., Bozeman, MT, USA) coupled to a double-focusing sector field ICP-MS (Spectro, Ametek, Kleve, Germany) with a Mattauch-Herzog geometry. It allows the simultaneous measurement of the mass range of *m*/*z* 6 to 238. Daily performance and fine tuning of the ICP-MS was performed with a NIST610 glass reference material (SRM-610, LGC Ltd., Middlesex, UK). ^12^C, ^13^C ^31^P, ^34^S, ^39^K, ^79^Br, and ^81^Br were the measured isotopes. Determination of both Br isotopes can have contributions from polyatomic interferences, especially the ^81^Br isotope by an abundant argon polyatomic ion [36]. Therefore, only the ^79^Br is shown in the results part. Zebrafish embryos were ablated in line scan mode with a chosen 50 μm spotsize (square shape) in order to be able to quantify Br at different exposure durations and to have sufficient spatial resolution. A second ablation was performed to ensure the completeness of the ablation of embryo. A mean of 4 ± 3% of the total ^12^C intensity was ablated during the second run. Furthermore, the ^28^Si signal was analyzed as marker for complete ablation. Parameters for the measurements are listed in Table S1 (see the ESM). The acquisition time of the ICP-MS together with the spotsize and scan speed of the laser allow the calculation of a calibration range per spot (50 × 50 μm). Data analysis was performed with Iolite 3.6 in Igor Pro 7.04. Exported data were overlaid with the optical photograph in MATLAB R2015b.

#### Neb-ICP-MS and IC

Parameters for neb-ICP-MS can be seen in Table S1 (see the ESM). Matrix-matched calibration (range from 10 to 100 μg/L bromide) was performed by taking 200 μL of MeOH extracts from negative control zebrafish embryos, adding a bromide standard solution and diluting to 4.2 mL with Milli-Q water (LOD 1.2 μg/L; LOQ 4.0 μg/L).

The IC experiments were performed with an ICS-2000 (Dionex, ThermoFisher Scientific) and an IonPac AS18, AG18, 4 mm column (Dionex, ThermoFisher Scientific). The eluent was 21–40 mM KOH in an eluent generator cartridge, a flow rate of 1 mL/min was applied and an injection volume of 5 μL. The temperature of the column was 30 °C. Before entering the conductivity detector, a suppressor (ASRS 300 4 mm) was used.

#### LC-MS/MS

LC-MS/MS measurements were performed with a 1260 Infinity HPLC system (Agilent Technologies, Böblingen. Germany) and a QTrap 5500 mass spectrometer (AB Sciex, Darmstadt, Germany, TurbolonSpray interface). An Ascentis® Express C18-column (10 cm × 3.0 mm; 2.7 μm; Sigma-Aldrich) was used and Milli-Q water with 10 mM NH_4_Ac as solvent A and MeOH with 10 mM NH_4_Ac as solvent B. The following gradient was applied: 0 min, 95% solvent A; 3 min, 5% solvent A; 6.1 min, 95% solvent A. The flow rate was 300 μL/min and 5 μL were injected. The calibration was performed by diluting naled and dichlorvos with MeOH (LOD: dichlorvos 2 nmol/L, naled 0.3 nmol/L; corresponds to dichlorvos 7 × 10^−2^ pmol/embryo, naled 8 × 10^−3^ pmol/embryo). The transitions for naled (398–127, 382–128) and dichlorvos (221–109, 221–127) were acquired in multiple reaction monitoring. Reported data for the extracts were not corrected for matrix effects and recovery after sample preparation (matrix effects naled < 45%, recovery naled < 5%; matrix effects dichlorvos < 10%, recovery dichlorvos > 90%).

#### Profilometer

Measurements were performed with a S neox non-contact 3D surface profiler (Sensofar, Barcelona, Spain). Data were analyzed with the software SensoScan 5.3 and SensoMap 7.0. Two profiles of two ablations, respectively, are depicted in Fig. S2 (see the ESM).

### Bromine quantification

The internal amount of Br was determined in three independent ways: (i) by summation of the calibrated ^79^Br-signal of the imaging data (LA-ICP-MS), (ii) in the extract of the homogenized embryos by Neb-ICP-MS, and (iii) in the extracts by IC as bromide. For LA-ICP-MS, the quantification was performed with agarose gels spiked with a bromide ICP-MS standard solution [30]. The standards were ablated under the same measurement parameters as the zebrafish embryos. The homogeneity of the standards was determined by ablating the total length and width of the glass slide: a decrease in the Br intensity was observed at the edges of the slide leading to a higher concentration in the middle. Therefore, a factor of 1.32 was applied during Br quantification to correct for this concentration gradient [30]. The workflow in Iolite 3.6 included a baseline subtraction, a correction of peak drift over time measured with the agarose standards, and the quantification of counts per second to mass. The calibration range was 37 to 598 ng Br/spot (two calibration curves are shown in ESM Fig. S3). After ablation of 4 embryos, a calibration was performed. This was necessary because the intensity of one agarose standard showed a high variation between different ablation cycles (incl. different days of ablation), e.g., 30,900–57,500 cps (RSD of 21%) for the agarose standard 62 pg Br/spot and 46,800–122,600 cps (RSD of 28%) for the agarose standard 108 pg Br/spot. A range for the LOQ was obtained of 38 pg Br/spot (calculated out of a signal to noise ratio of 10 in a blank agarose standard). In order to account for the varying ablated mass of the dried embryo, the intensity ratio of ^79^Br to ^12^C was calculated.

## Results and discussion

### Time course of uptake and effect during exposure of zebrafish embryos to naled

**Fig. 1:**
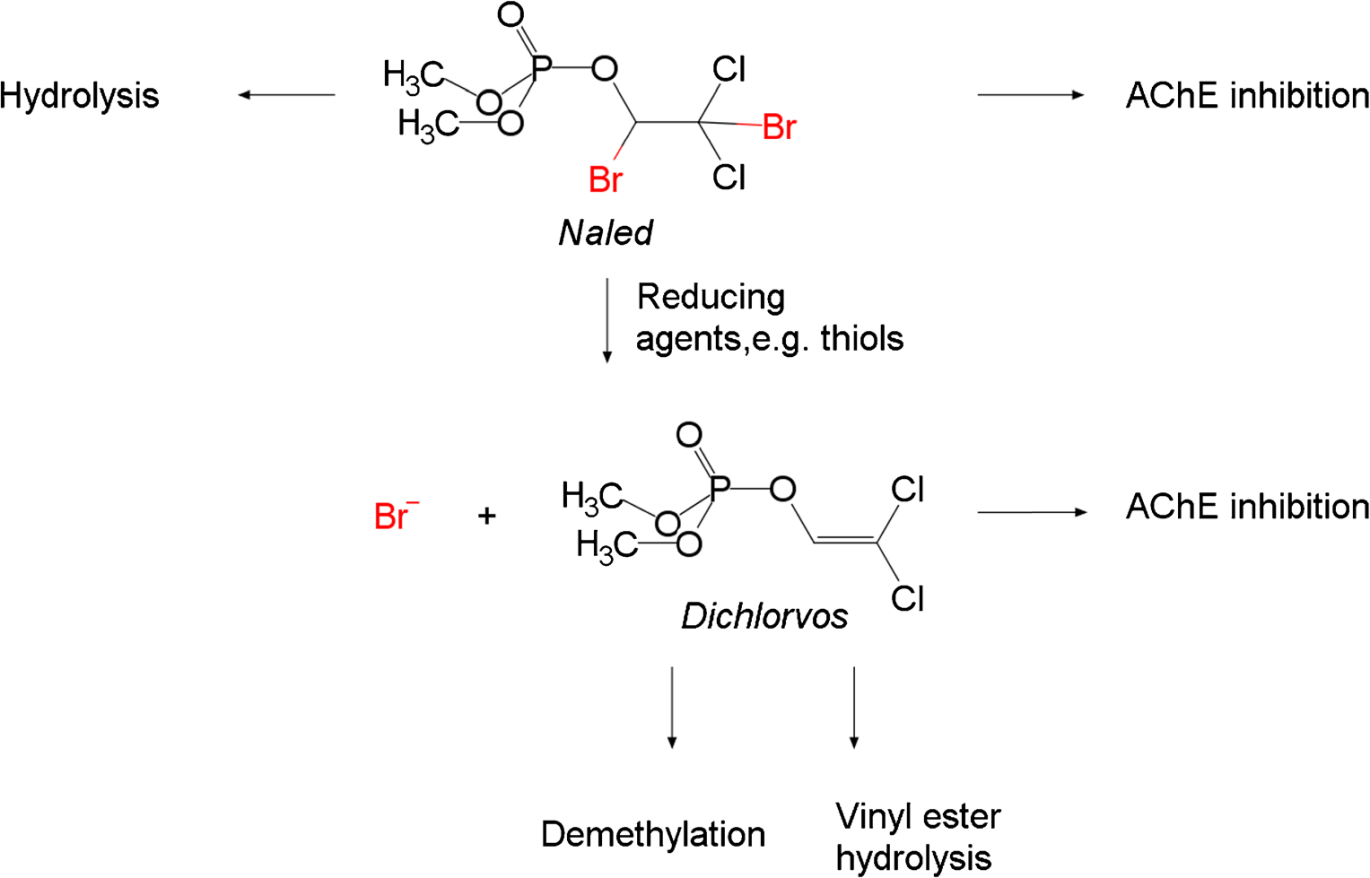
Transformation products and biological effects of naled. Naled may be hydrolyzed (mainly in the exposure solution) or reduced to dichlorvos (in biological systems). Total Br in the tissue was quantified with LA-ICP-MS and in extracts by neb-ICP-MS, naled and dichlorvos were determined in extracts by LC-MS/MS, bromide by ion chromatography. AChE activity was determined in an enzyme assay

Naled as a phosphoric acid triester is an electrophile that reacts with AChE or with other nucleophiles including water (Fig. 1). Moreover, naled has been reported to undergo non-enzymatic reductive debromination in biological tissues to form dichlorvos, which can also act as AChE inhibitor [37–39]. Dichlorvos may be further transformed to non-toxic and non-brominated products. It should be noted that during the time of exposure the external concentration of naled decreased from 3.7 to 1.0 μmol/L (ESM Fig. S4), likely due to hydrolysis [37]. The hydrolysis products of naled do not act as AChE inhibitors and are more hydrophilic. Hence, they are possibly taken up more slowly than the parent compound [40]. Because naled is highly reactive, its uptake and distribution in the embryo may not be determined by analyzing naled itself. Indeed, naled could not be detected in embryo extracts by LC-MS/MS (LOD: 8 fmol/embryo) at any time during exposure to 3.7 μmol/L naled.

**Fig. 2:**
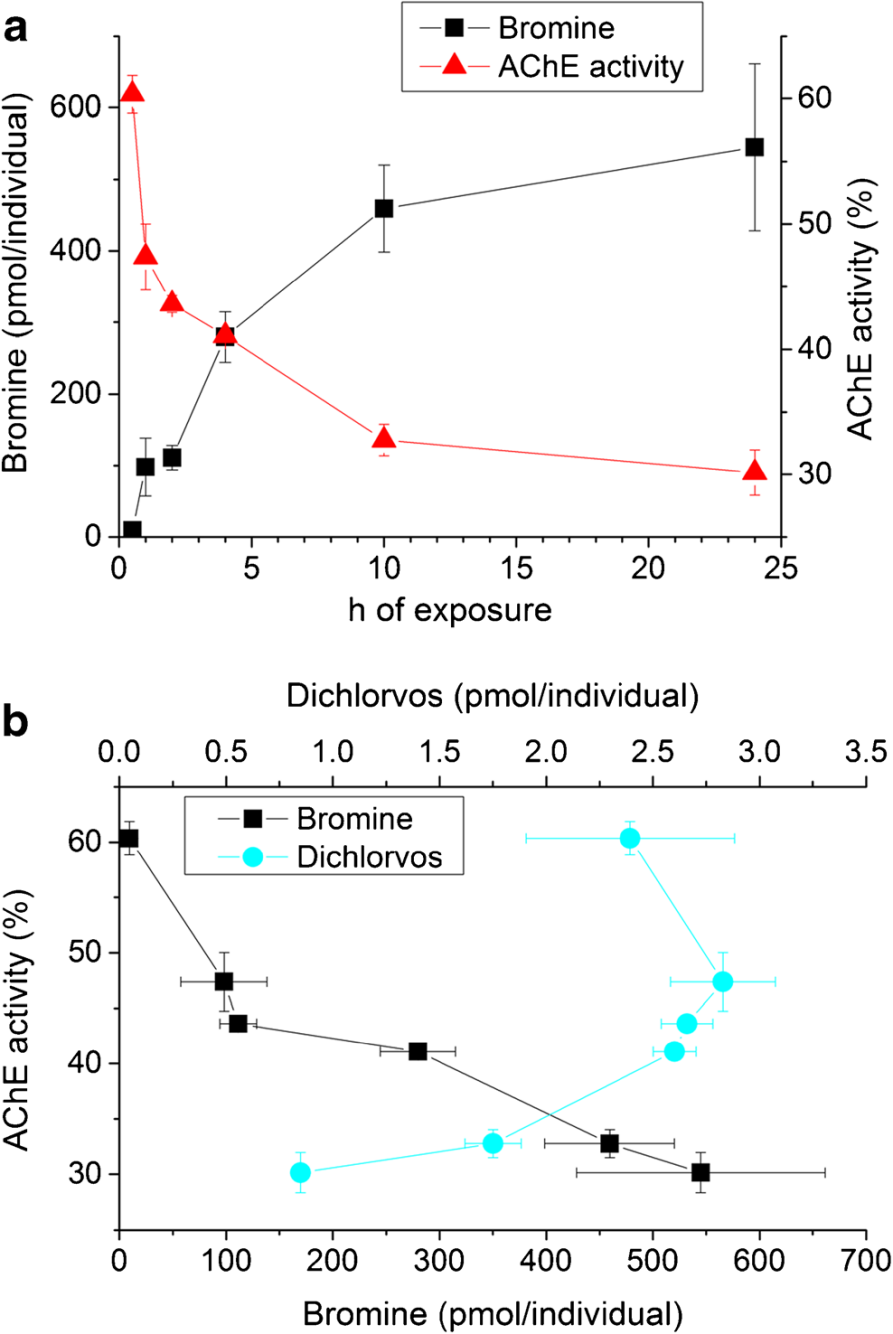
**a** AChE inhibition (*n* = 3) normalized to the specific activity in the negative control and integrated Br amount in the zebrafish embryo (*n* = 4, in the case of 24 h *n* = 5) determined with LA-ICP-MS. **b** AChE activity plotted against the internal dichlorvos (LC-MS/MS, *n* = 3) and Br amounts (measured with LA-ICP-MS) (see also ESM Fig. S5). Endpoint of the exposures was 96 hpf

However, it was hypothesized that the amount of Br in the embryo reflects the amount of naled taken up. Indeed, the internal Br amount rapidly increased within the first 10 h of exposure to naled and reached 545 pmol/individual after 24 h exposure (Fig. 2a). Based on a volume of 250 nL for the zebrafish embryo at 96 hpf (ESM Fig. S6), this Br amount would correspond to a 280-fold enrichment of naled in the embryo compared to the exposure solution, provided that all Br in the embryo originates from naled. This would be two orders of magnitude higher compared to the bioconcentration factor in zebrafish embryos reported by Brox et al. for compounds with a similar octanol-water partition coefficient (e.g., phenacetin (log *D* = 1.41), colchicine (log *D* = 1.46)) [12]. Thus, the transformation of the parent compound to the bromine-containing transformation product results in an accumulation of Br. No significant decrease of the Br signal was visible over 24 h of depuration (*t* test: *p* = 0.22 for 1 h depuration and *p* = 0.55 for 24 h depuration, respectively) (ESM Fig. S8).

AChE activity in the embryos decreased with increase in Br amount and after 24 h exposure was at 30% compared to the non-exposed control (Fig. 2a). Given that AChE inhibition quickly proceeds [41], the inverse time course of the internal Br amount and AChE activity suggests a rapid distribution of naled or of its transformation product to the target site AChE. As there is no apparent time delay between the acquirements of measurable tissue levels and the AChE inhibiting effect of naled in the organism, the internal Br amount may, therefore, be a suitable measure for the biological effect amplitude.

LC-high-resolution MS (LC-HRMS) analysis of the embryo extracts (ESM Table S2) was performed and the data were searched for signals of other brominated or chlorinated transformation products of naled. However, there were no indications for the presence of other brominated compounds. Additionally, the bromide amount in embryo extracts was analyzed by IC, showing that the Br detected by LA-ICP-MS, indeed, occurred as bromide (Table 1). With respect to toxicokinetics, these findings consistently suggest that the entire naled that initially was taken up by the embryo was quantitatively transformed to non-brominated products, among them dichlorvos and bromide. The Br amounts were well above the sum of internal amounts of naled and dichlorvos, e.g., 2 times as high at 0.5 h exposure and 326 times higher at 24 h.

**Table 1:** Comparison of internal amounts of Br determined by LA-ICP-MS (*n* = 4, in the case of 24 h *n* = 5), neb-ICP-MS (*n* = 3), and of bromide measured with IC (*n* = 3) (mean in pmol/individual and %rsd)

h of exposure Endpoint: 96 hpf	Br LA-ICP-MS		Br in extracts neb-ICP-MS		Bromide in extracts IC		Br: LA-ICP-MS/neb-ICP-MS	Br vs. bromide: LA-ICP-MS/IC
	pmol/individual	%rsd	pmol/individual	%rsd	pmol/individual	%rsd		
0.5	9.3	24	41	50	< LOQ		0.23	< LOQ IC
1	98	38	90	11	58	30	1.1	1.7
2	111	15	167	2	106	11	0.66	1.0
4	280	13	342	5	232	10	0.82*	1.2
10	459	13	507	6	398	6	0.91	1.2
24	545	21	713	7	462	16	0.76*	1.2

* Labeled ratios indicate that the mean of the two methods are significantly (Welch’s *t* test or Mann-Whitney *U* test, *p* < 0.05) different

The time course of the internal amount of dichlorvos is not correlated to the AChE inhibition, whereas the internal Br amounts significantly correlate with the AChE inhibition (Fig. 2b; Spearman correlation *p* = 0.18 and 0.0028, respectively). This may indicate that irreversible binding of naled to AChE proceeds more quickly than the transformation of naled to dichlorvos, so that the internal dichlorvos amount is not relevant for the biological effect. This would contradict the earlier assumption from a rat and mouse study that indeed dichlorvos is the biologically effective form of naled [39, 42]. Since the inhibition of AChE is due to binding to the AChE and transesterification of the toxicant, either naled or dichlorvos, the time course of the internal amount of dichlorvos may not be expected to represent the time course of the inhibition. In addition, the concentration gradient of dichlorvos to the exposure solution may also affect a possible correlation with the AChE inhibition. Hence, internal Br amount can be used as an indicator of the uptake of naled but does not help to clarify the mechanism of the subsequent inhibition of AChE.

The internal amounts of Br were also determined from ZFE extracts by neb-ICP-MS. For most sampling time points, they were higher than those calculated from the spatially resolved LA-ICP-MS results (Table 1). The internal Br amounts were particularly underestimated by LA-ICP-MS after short exposure periods when internal concentrations are lowest: e.g., at 0.5 h of exposure only 23% of the amount determined in the extract by neb-ICP-MS was found by LA-ICP-MS. If tissue concentrations are low, the signal intensity of most spots analyzed by LA-ICP-MS is below the LOQ, whereas the total concentration is well measurable from the extract by neb-ICP-MS. At Br levels of 100 pmol/individual, the agreement of Br quantities is acceptable (66–110%): this confirms previous findings that LA-ICP-MS imaging can be performed quantitatively if combined with external calibration using spiked agarose gels [30]. The bromide data in Table 1 support the validity of both quantitative approaches, one consisting of air-drying of the embryos followed by LA-ICP-MS, the other one of extraction of the embryos, solvent change, and IC analysis.

### Validation of the LA-ICP-MS measurements for the ablation of biological tissue of varying thickness

In this study, the entire air-dried embryo was ablated rather than a slice of constant thickness as would be obtained by sectioning. On the one hand, this may have several disadvantages. (i) The visual image of the signal intensity of an element does not represent its (variable) concentration as the mass of the biological material ablated per spot is not constant but varies with the thickness of the object at that site. However, the completeness of the ablation was ensured by a second ablation (see the “Material and methods” section). (ii) Also, the amount of matrix may increase with increasing thickness of the object. However, this was found to be negligible when validating the quantification with the neb-ICP-MS. (iii) Smaller organs of the embryo, e.g., its liver or heart may be less clearly visible if they are superimposed by other body compartments. On the other hand, LA-ICP-MS imaging of the whole air-dried embryo provides advantages: it provides higher sensitivity as the whole organism is available for analysis rather than a slim tissue section, only, it excludes the uncertainty of extrapolating quantitative data obtained from a limited number of cryosections to the whole body and it requires less sample preparation.

Due to the variable thickness and tissue density of the embryo, the Br intensity images need to be normalized to the amount of biogenic organic matter ablated at each spot to obtain Br concentration images. Here, ^12^C was used for this purpose, as its concentration should be fairly uniform among the different biological tissues [43].

The ^12^C signal intensities were partly comparable with the thickness profile recorded for three embryos before the laser ablation process by a profilometer (ESM Fig. S2). The ^12^C to thickness ratio of the data normalized to a range from 0 to 1 was in average 1.1 with a rsd of 51% (for 269 spots from 9 transects of three ablations). The element ^31^P signal exhibited a similar ratio and could be used for the normalization as well (ESM Fig. S2) [18, 24, 44]. In the region of the swim bladder, the ^12^C signal intensity and the thickness were deviating. Since the swim bladder represents a gas-filled organ, the thickness does not correlate to the tissue amount. Moreover, from the head of the embryo the intensity of ^12^C increases to the tail (see an example for both observations in ESM Fig. S2i). The latter may be related to the higher tissue density in the yolk of the embryo which may lead to larger particle sizes generated by the laser ablation, a diminished particle transport efficacy to the plasma, and, therefore, a lower sensitivity of the ICP-MS [45]. This may also contribute to the deviation of the LA-ICP-MS and neb-ICP-MS results. A further advantage of normalization to the intensity of ^12^C is the possibility to compensate for instrumental fluctuations during LA-ICP-MS.

### Spatial distribution of Br in the zebrafish embryo investigated with LA-ICP-MS

**Fig. 3:**
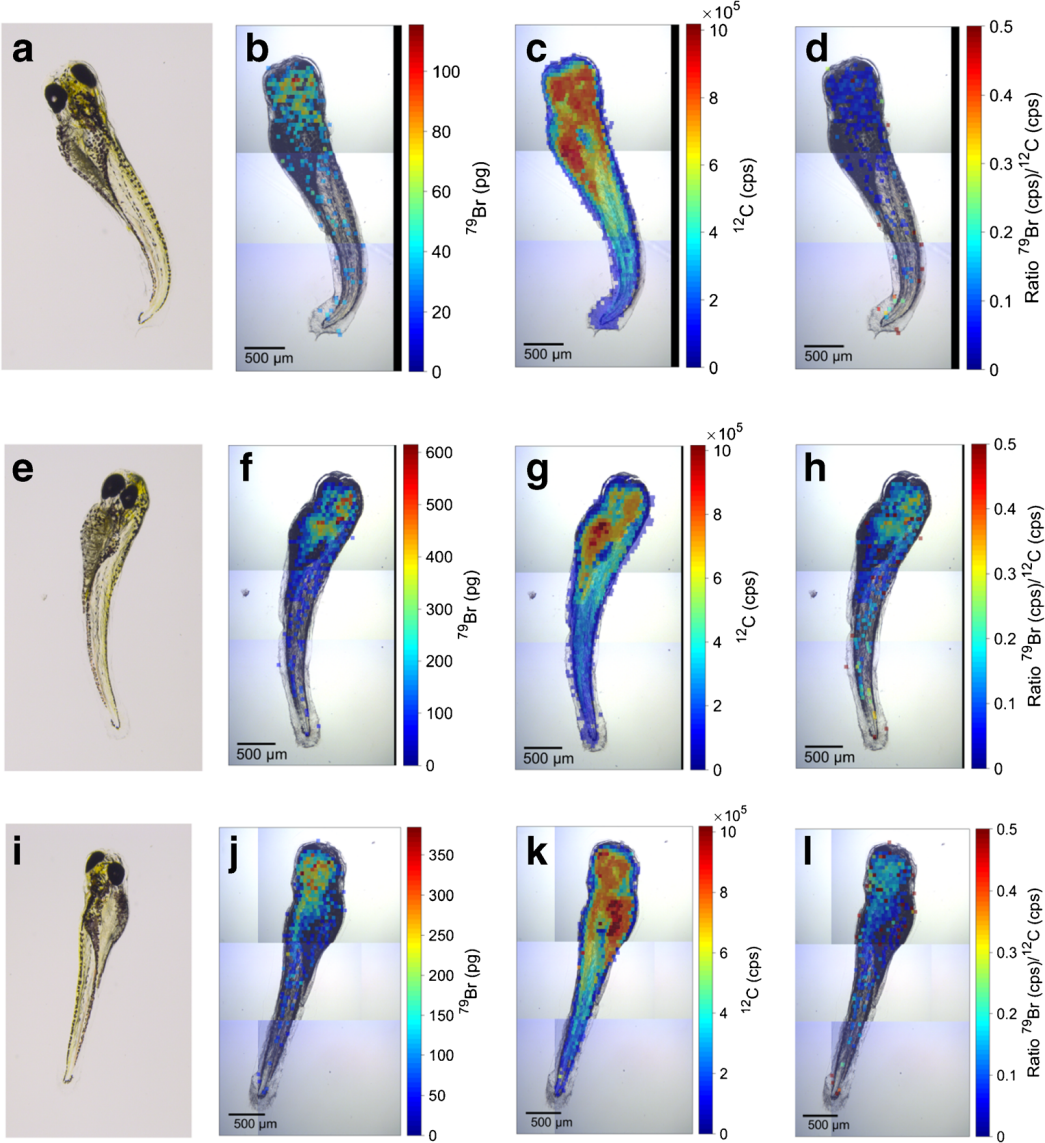
Microscopic images of the zebrafish embryo (**a**, **e**, **i**) and spatially resolved images obtained by LA-ICP-MS for ^79^Br (**b**, **f**, **j**), ^12^C (**c**, **g**, **k**), and the ratio of intensities (cps) of ^79^Br to ^12^C (**d**, **h**, **l**). A spotsize of 50 μm was used for ablation. Intensities that were below the LOQ were not color coded. Zebrafish embryos were exposed for 2 h (**a**–**d**) and 24 h (**e**–**h**); endpoint was 96 hpf. Zebrafish embryos in **i**–**l** were exposed for 24 h with 24 h depuration (endpoint was 120 hpf)

The total internal amount of a compound does not provide information on its spatial distribution in the organism and how much of the compound is present in the tissue areas with the biological target site. Therefore, the distribution of Br in the zebrafish embryo was determined by quantitative LA-ICP-MS analysis, after different durations of exposure (Fig. 3b, f, j). The color-coded visual images of the Br distribution showed the most intensive Br signal in the head region already after 2 h of exposure (Fig. 3b). This distribution appears to remain stable or to become even more pronounced until the end of the exposure (24 h, Fig. 3f) and remains also after 24 h of depuration (Fig. 3j). The portion of Br in the head region corresponds to approximately 40% of the total Br (calculated by defining regions of interest with “Monocle” in Iolite, Tables 1 and 2) [46]. This is consistent over the different exposure duration. An enrichment of Br is not seen for the yolk, although this compartment accounts for a similar portion of the body mass (29% of the total carbon as mean of the different exposure durations, in contrast the head accounts for 26% of the total carbon).

**Table 2:** Total Br amounts and mean ^79^Br/^12^C ratios in the regions of interest head and yolk measure with LA-ICP-MS (an example for the definition can be found in ESM Fig. S7). Endpoint of exposure was 96 hpf. (0.5 h exposure are not displayed because of the low Br amount)

h of exposure	Br head pmol/individual	%rsd	Br yolk pmol/individual	%rsd	^79^Br/^12^C head	%rsd	^79^Br/^12^C yolk	%rsd
1	32	14	5.4	36	0.019	11	0.0048	40
2	54	17	12	35	0.042	8.1	0.0088	24
4	102	9.3	45	21	0.048	20	0.015	27
10	168	12	75	34	0.10	18	0.036	20
24	216	25	117	25	0.12	15	0.047	18

The mean ^79^Br/^12^C ratio confirms the enrichment of Br in the head region, exhibiting levels between 2.6 and 4.7 times higher than in the yolk (Fig. 3d, h, l) (Table 2). However, Br amounts were often below the LOQ of the LA-ICP-MS approach, namely for the thin tail and also partly for the yolk. Therefore, ^79^Br/^12^C ratios could only be calculated for 30% of the spots.

### Evaluation of the uncertainty of the LA-ICP-MS measurements and biological variation

Independent from the normalization, a visual inspection and comparison of images could be subjective and prone to misinterpretation. A visual inspection does neither provide a measure for the similarity of the elemental distribution of individuals in one experiment (i.e., the precision) nor for the differences between the mean distributions of different exposures.

Obviously, a quantitative measure is needed to describe the degree of homogeneity in the spatial distribution of an analyte in imaging mass spectrometry. This measure would allow comparing both the similarity in the distribution of an analyte in biological replicates as well as the dissimilarity in the distribution between organisms exposed under different conditions. On this basis, it would be possible to judge as to whether differences in the spatial distribution between groups of organisms are statistically significant.

**Fig. 4:**
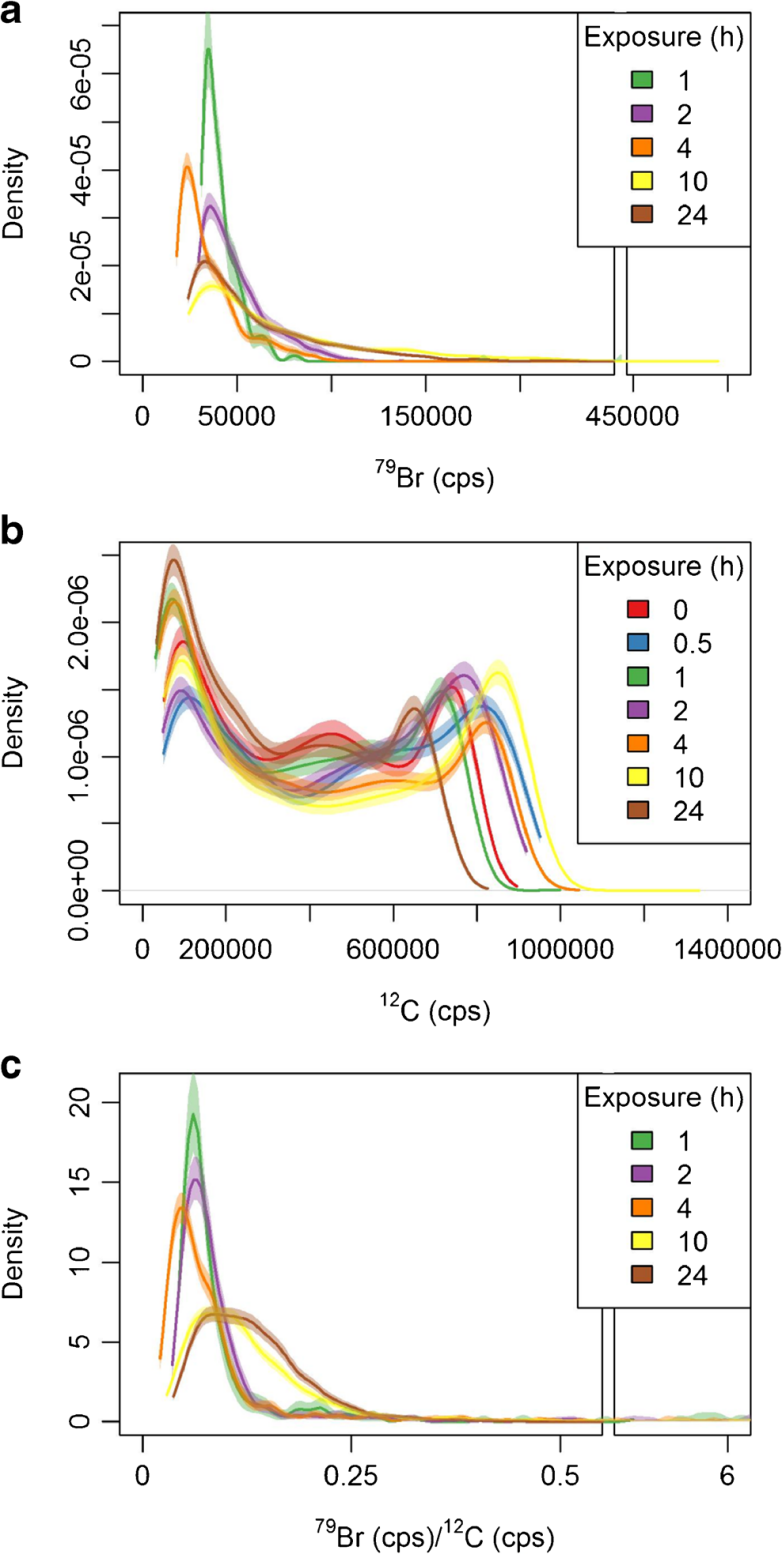
Kernel density estimates with 5% confidence interval (using the density function in R3.3.2 with default smoothing settings, confidence intervals were calculated with 1000 bootstrap iterations) for **a**^79^Br, **b**^12^C, and **c**^79^Br/^12^C; *n* = 4 for each exposure duration (except 24 h exposure *n* = 5). At 0 and 0.5 of exposure to naled, only few pixels contained Br intensities above the LOQ restricting the calculation of Kernel densities

For this purpose, Kernel density estimates of the intensities per pixel were calculated from the LA-ICP-MS data of the zebrafish embryos (Fig. 4). Kernel density estimates have been conducted previously for an experimental-modeling-approach in LA-ICP-MS and as a first visual inspection tool [46, 47]. However, to our knowledge Kernel density estimates have not yet been used for a comprehensive comparison of the distribution of toxicants between biological objects.

In the Kernel density estimates, the width of the confidence interval of the density estimates, i.e., the width of the lines in Fig. 4, are influenced by the measurement uncertainty as well as by biological variability between individual organism and is, thus, a measure of the total precision. For ^79^Br, the lines are very narrow and significant differences between the different times of exposure are visible (Fig. 4a). For ^12^C, the lines are wider and less distinct, indicating a higher variability in the ^12^C distribution of individuals (Fig. 4b).

The distribution of ^79^Br in the zebrafish embryos is monomodal and positively skewed (Fig. 4a). In contrast, Kernel density estimates of the ^12^C intensities are bimodal (Fig.4b). The thicker parts of the embryo (head and yolk) are represented by the maximum at higher and thinner parts of the embryo by the maximum at lower intensities. By comparing Fig. 4a and Fig. 4b, it becomes clear that ^79^Br and ^12^C are distributed differently. Theoretically, an even distribution of ^79^Br and ^12^C would be represented by a vertical line in the graph for the ^79^Br/^12^C ratio, while real, measured data of the even distribution would translate into a (narrow) Gaussian curve due to measurement uncertainty. The difference between biological replicates would broaden this Gaussian curve further. Individual density curves for the replicates can be seen in Figs. S9 and S10 (see the ESM).

The density curves of ^79^Br/^12^C in Fig. 4c show a change in distribution from a narrow (more homogenous) distribution at 1 h (green line in Fig. 4c) towards a broader distribution with increasing frequency of spots with higher ^79^Br/^12^C ratios after 10 h (yellow line). The 24 h density (brown line) is comparable to the 10 h. This is consistent with the stable total internal Br amount in this period of exposure (Fig. 2a). The effect of the exposure duration is particularly visible in the density curves of ^79^Br/^12^C in Fig. 4c stressing the importance of the normalization.

The Kernel density estimates turn out to be a useful tool to exploit the data on Br concentrations resolved in space and time.

### Toxicokinetics of naled based on time-resolved imaging data

The LA-ICP-MS imaging of zebrafish embryos after different durations of exposure to naled combined with a suite of complementary analytical techniques allows drawing the following picture of the toxicokinetics of naled:

The internal amounts of Br (Fig. 2 and Table 2) show that naled is taken up and rapidly quantitatively transformed to bromide and smaller amounts of dichlorvos and other possible transformation products. This transformation possibly increased the uptake and lead to a strong enrichment of Br in the embryo. After 10 h of exposure, the internal Br amount was stable for the rest of the exposure duration. The AChE inhibition correlates with the internal Br amount (Fig. 2a). Br in the embryo occurred as bromide, as shown by IC.

LA-ICP-MS imaging confirmed an enrichment of Br in the head compared to the yolk of the embryos (Fig. 3). Bromide may have been released from naled at the target site before or after covalent binding to and inhibition of the AChE. However, the Br enrichment in regions rich in nervous tissue and, hence, in cholinergic synapses, suggests that naled was accumulated and reacted with its target, possibly releasing dibromdichloroethanol which was eventually debrominated. The local accumulation may be explained partially by affinity to the target site. This hypothesis is supported by literature. Firstly, it has been shown for AChE in other fish (electric eel) that naled is more reactive towards AChE than dichlorvos, with a bimolecular rate constant being 17 times higher [41]. Secondly, it has been shown by staining that AChE is located in the brain and heart of zebrafish embryos [48, 49].

Alternatively, bromide may have been released at the target site prior to binding of a transformation process such as dichlorvos to the AChE. Then the distribution of Br would indicate higher frequencies of the reducing agents that transform naled to dichlorvos (e.g., thiols) in these regions. Subsequently, dichlorvos rather than naled reacts with and thereby inhibits the AChE. This was suggested by Casida (1972). However, in this case one may not expect an accumulation of Br in regions rich of the AChE target. The rapid transformation could be tested by detection of thiols representing major reductants of naled. It could be expected that thiols or sulfur is enriched in the same region as bromide. However, the ^34^S signal obtained by our LA-ICP-MS analyses was too weak to generate a meaningful image of its distribution in the embryo. Also, a parallel occurrence of both processes—covalent binding of naled and its transformation products—may be possible.

## Conclusion

This study demonstrated that the combined time-resolved analysis of biological activity, of total internal concentrations and of the tissue distribution of naled in zebrafish embryos by LA-ICP-MS, in combination with additional analytical techniques allows to elucidate the toxicokinetic and initial steps of the toxicodynamics of this reactive toxicants. It was demonstrated for the AChE inhibitor naled that these processes can be quite complex. Quantitative imaging by LA-ICP-MS is attractive to determine the spatial distribution of toxic compounds with a suitable elemental signature and to detect their enrichment in certain regions of the zebrafish embryo. Beyond Br also other heteroatoms such as iodine, arsenic, platinum, or gadolinium can be found in chemicals released by human activity to the environment. For these compounds, LA-ICP-MS represents a suitable method to study uptake and distributions in the zebrafish embryo model.

Compared to the imaging of sections of zebrafish embryos, the analysis of air-dried whole embryos is much easier. If combined with normalization to ^12^C, it allows for quantitative imaging and the determination of concentrations in selected regions of the embryo. Kernel density estimates are helpful tools to assess the homogeneity of the distribution for elements or molecules determined by MS imaging in the zebrafish embryo and to assess its variability between individuals. They are essential also to determine differences in the internal distribution over time. This may be further developed, e.g. by parametrization of the density estimates, in order to statistically show the significance of the exposure time or distribution of different elements.

## Electronic supplementary material

ESM 1 (PDF 826 kb)
