# Supplementary material for: Elemental imaging (LA-ICP-MS) of zebrafish embryos to study the toxicokinetics of the acetylcholinesterase inhibitor naled
Source: Anal Bioanal Chem. 2018 Nov 16;411(3):617–27. doi: 10.1007/s00216-018-1471-2 (PMC6338705; doi:10.1007/s00216-018-1471-2)
Supplement: Supplementary file 1 — (PDF 826 kb) [file 216_2018_1471_MOESM1_ESM.pdf]

Analytical and Bioanalytical Chemistry

Electronic Supplementary Material

Elemental imaging (LA-ICP-MS) of zebrafish embryos to study the toxicokinetics of the acetylcholinesterase inhibitor naled

Katharina Halbach, Stephan Wagner, Stefan Scholz, Till Luckenbach, Thorsten Reemtsma

AChE activity measurements at different naled concentrations

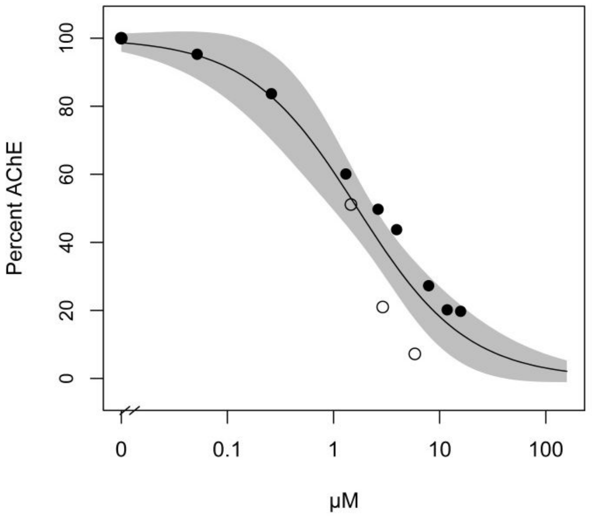

Fig. S1 AChE activity presented as percentage of the activity of the control measured in zebrafish embryos exposed to different concentrations of naled from 72 to 96 hpf. (Data points are the mean of three technical replicates; filled and open symbols represent two independent exposure experiments). The line represents the modelled concentration-response-curve obtained using a sigmoidal model and using the R package drc. EC50=1.68 (0.961-2.4 confidence interval); slope= -0.843

Instrumental parameters of LA-ICP-MS and neb-ICP-MS measurements

Table S1 LA-ICP-MS and nebulization-ICP-MS parameters

		Neb-ICP-MS	LA-ICP-MS
ICP-MS			
RF power	[W]	1550	1300
Cooling gas flow rate, Argon	[L/min]	12	13.50
Auxiliary gas flow rate, Argon	[L/min]	2.30	2.6
Nebulizer gas flow rate, Argon	[L/min]	0.88	/
Carrier gas flow rate, Helium	[L/min]	/	0.8
Integration time	[s]	10	1
Baseinterval	[ms]	1000	100
Laser ablation system			
Wavelength of ArF laser	[nm]		193
Laser beam diameter	[µm]		50
Laser scan speed	[µm/s]		50
Repetition frequency	[Hz]		100
Laser fluence	[J/cm^2]		1.79 (25% laser output at 4 mJ)

Profilometer data

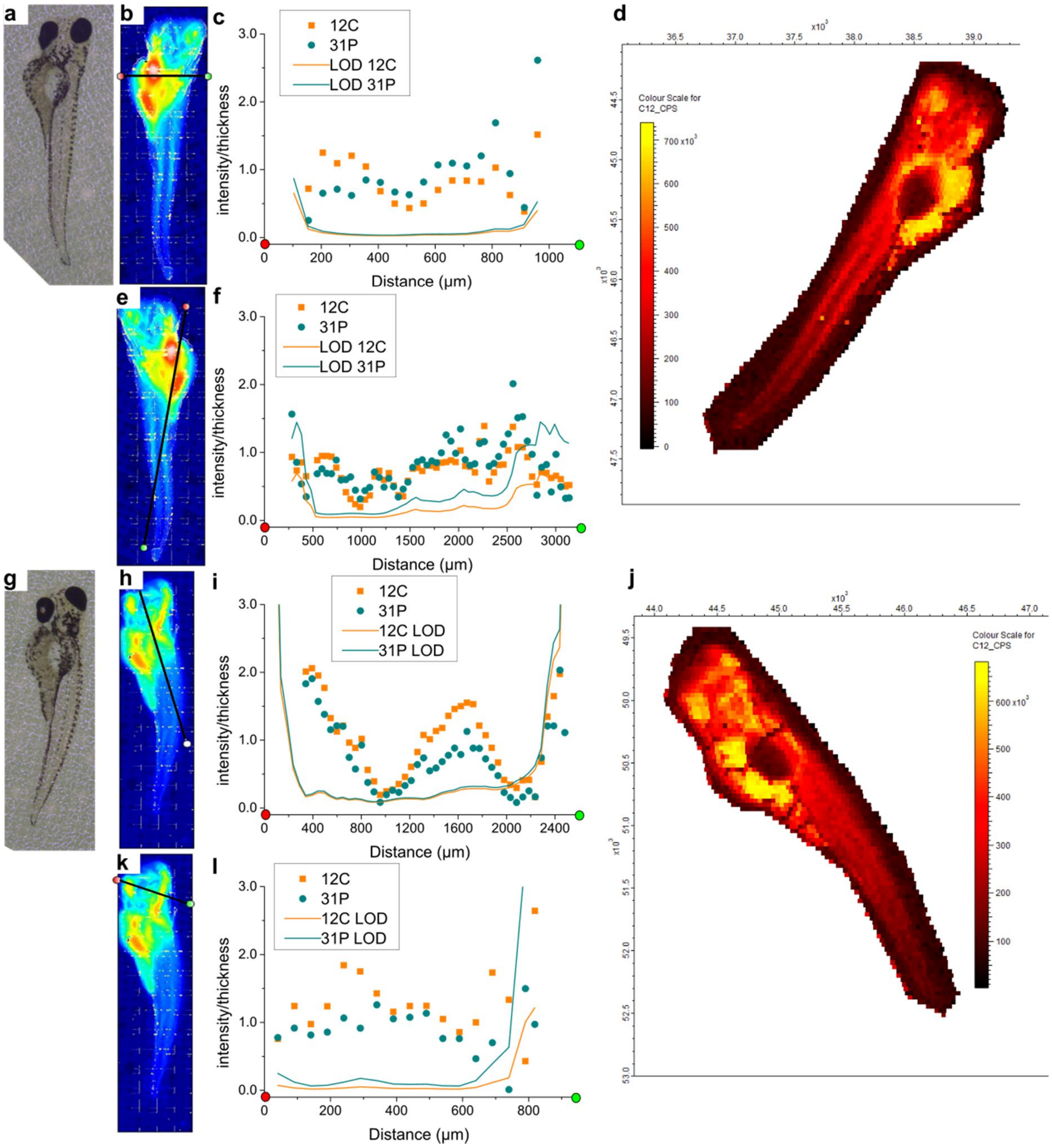

Fig. S2 Profilometer data before ablation and corresponding intensities of ^{12}C, ^{31}P, ^{39}K after ablation with LA-ICP-MS of 96 hpf zebrafish embryos. **a**, **g:** Microscopic image; **b**, **e**, **h**, **k**: Heat map of thickness measurement, line indicates the linear profile (red is the starting point, green the end point); **c**, **f**, **i**, **l**: Scatter graph of the ratio of the intensities (counts per second) of LA-ICP-MS measurement (spot size 50 µm) and the thickness (µm) of the profile; lines, respectively, indicate the ratio (intensity/thickness) belonging to the LOQ ($32.5 \cdot 10^3$, $22.3 \cdot 10^3$cps for ^{12}C, ^{31}P, respectively) of the elements; **d, j**:intensity (cps) map of the ^{12}C signal measured with LA-ICP-MS. The intensity and thickness data were scaled to a 0-1 range in order to calculate the ratio

Calibration with agarose gels for LA-ICP-MS

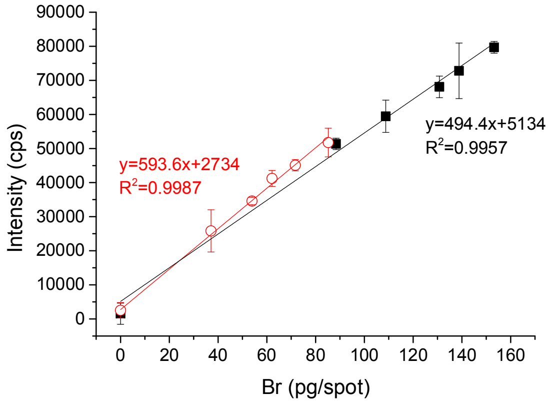

Fig. S3 Two examples for measured calibration curves in different concentration ranges, spotsize: 50 µm. Calibration curves conducted for experiments with embryos exposed from 86 to 96 hpf (black square) and from 95 to 96 hpf (red circle)

LC-MS/MS of aliquots from exposure solution

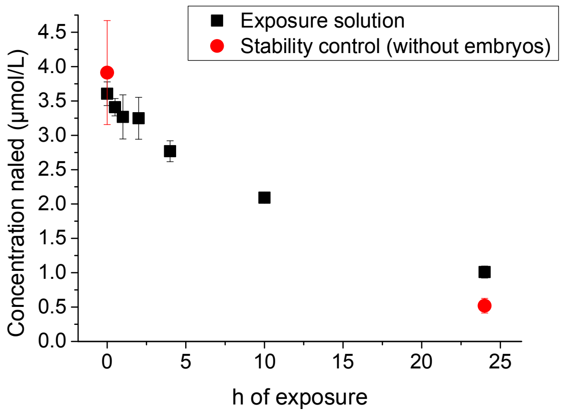

Fig. S4 Concentration of naled in the exposure medium (n=3) decreased with exposure time due to hydrolysis

Time course of dichlorvos internal concentration

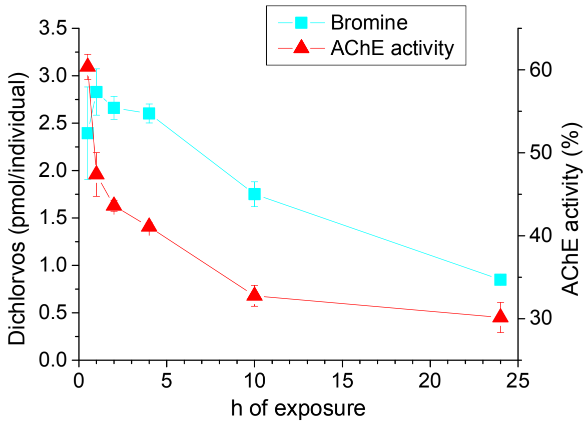

Fig. S5 AChE inhibition (n=3) normalized to the specific activity in the negative control and internal dichlorvos amount in the zebrafish embryo (n=3) determined with LC-MS/MS

Zebrafish embryo and embryo volumes

Zebrafish embryo volumes of different stages were estimated using the VAST Bioimager (Union Biometrica, Holliston, MA, USA). For each stage images of 10 embryos were taken in dorsal, ventral and lateral (left, right) position. Images were loaded into a KNIME workflow with appropriate image processing nodes [1]. On each image the trunk, yolk and pericardium were labelled manually using the KNIME interactive image annotator. Subsequently, images were sliced virtually in 10 µm sections. Each section was considered as an ellipsoid cylinder for which the volume was estimated based on the lateral and dorsoventral diameter. The total volume was calculated by summing up the slice volumes. Volume for 96 hpf stages were obtained by interpolation from mean volumes of stages used to estimate the volume (Fig. S6).

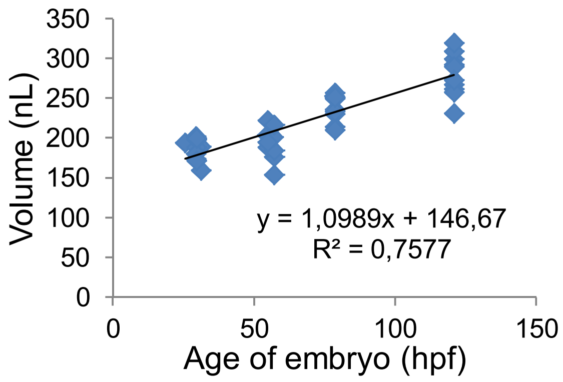

Fig. S6 Volumes of zebrafish embryos (incubated at 26 °C)

LC-HRMS measurements

A Xevo G2-XS Quadrupole-Time-of-flight (Waters, Milford, USA) coupled to a UPLC Acquity I-Class System (Waters, Milford, USA) equipped with an electrospray interface was used for the non-target screening. Separation was performed on a Waters Acquity UPLC HSS T3 (2.1x100 mm, 1.8 µm) at a column temperature of 45 °C. Water (A, pH 5, 10 mM ammonium acetate) and MeOH (B, 10 mM ammonium acetate) were used as eluents. 10 µL of the sample was injected and a total flow rate of 450 µL was applied. The gradient was as follows: 0.0 min, 2 % B; 0.25 min, 2 % B; 12.5 min, 99 % B; 13.0 min 99 % B; 13.01 min, 2 % B; 17.0 min, 2 % B. Further parameters for the ionization can be found in Table S2. Leucine enkephaline was used as lock spray reference. The mass range from m/z 50-1200 was scanned in positive and negative mode. The data was acquired with a low (4 eV) and a high collision energy (15-40 eV), in parallel. The data were analyzed with UNIFI (version 1.7; Waters).

Table S2 Parameters of the electrospray ionization for the UPLC-Tof measurements

Capillary voltage (kV)	0.8;-0.8
Source temperature (°C)	140
Desolvation temperature (°C)	600
Sampling cone voltage (V)	20
Source offset voltage (V)	80
Cone gas	Nitrogen
Collision gas	Argon

Definition of areas of interest with “Monocle” in Iolite

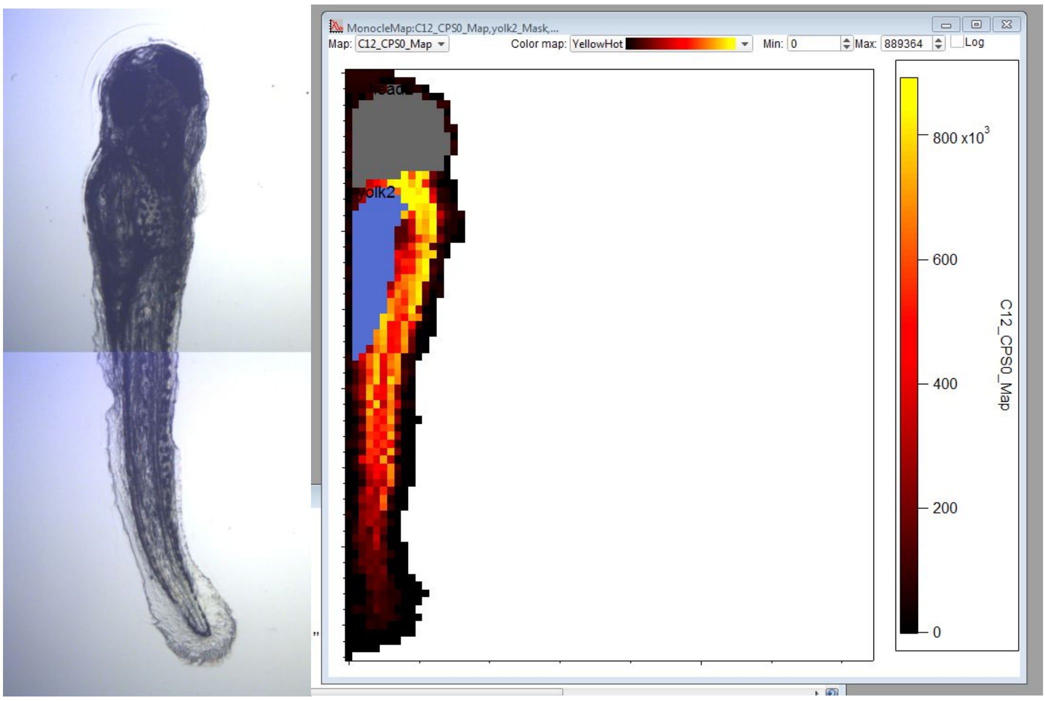

Fig. S7 Microscopic image and defined areas of interest in Iolite with “Monocle” [2] (grey: head; blue:yolk). Displayed is the ^{12}C distribution but the same defined areas of interest applies also for other measured elements or the calculated ratio (by simply changing the channel)

Depuration experiments

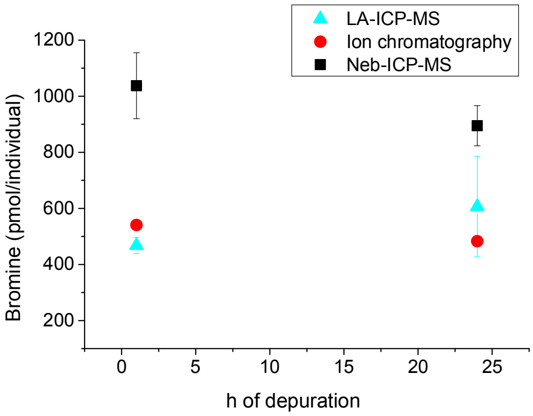

Fig. S8 Br and bromide (in case of ion chromatography) internal amounts after 1 and 24 h of depuration (exposure from 72 to 96 hpf)

Frequency distribution of LA-ICP-MS measurements

The Kernel density estimates of two individuals of an exposure duration of 4 h may contain outliers leading to the local maximum around and above 300000 cps (Fig. S10c). In order to identify these pixels as outliers one would also need to take the spatial distribution and the size of body compartments into account. Since it is reasonable that body compartment smaller than the spot size are present, these pixels were not excluded from the data analysis. Otherwise, the variation between biological replicates is comparatively narrow (rsd of the densities at the maxima are <23% except for the ^{79}Br signal at 1 h exposure, Table S3) which proves that the precision of the whole approach is high (Fig. S9 and Fig. S10).

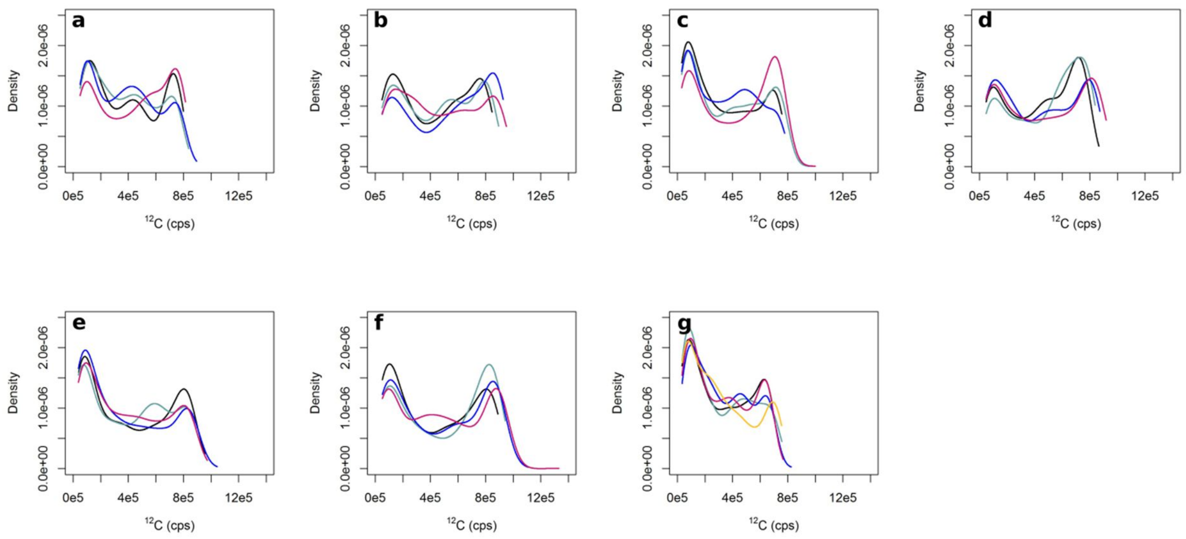

Fig. S9 Kernel density estimates of LA-ICP-MS intensity (cps) data for ^{12}C (using the density function in R3.3.2 with default smoothing settings). Pixels with intensities below the LOQ were removed. **a** control; **b** exposure from 95.5 to 96 hpf; **c** exposure from 95 to 96 hpf; **d** exposure from 94 to 96 hpf; **e** exposure from 92 to 96 hpf; **f** exposure from 86 to 96 hpf; **g** exposure from 72 to 96 hpf

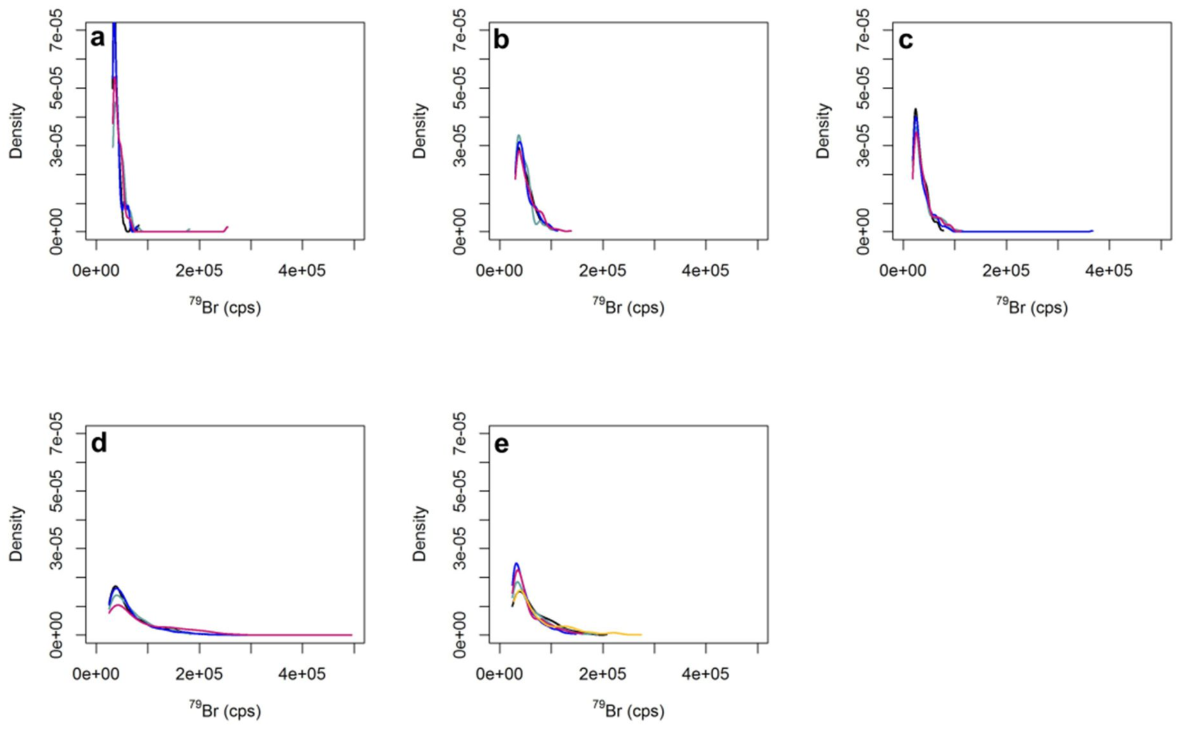

Fig. S10 Kernel density estimates of LA-ICP-MS intensity (cps) data for ^{79}Br (using the density function in R3.3.2 with default smoothing settings). Pixels with intensities below the LOQ were removed. **a** Exposure from 95 to 96 hpf; **b** exposure from 94 to 96 hpf; **c** exposure from 92 to 96 hpf; **d** exposure from 86 to 96 hpf; **e** exposure from 72 to 96 hpf

Table S3 Mean and relative standard deviation of the densities for the maxima in Fig. S9 and Fig. S10

Exposure duration	^{12}C		^{12}C		^{79}Br	
	Mean density maximum 1	%rsd	Mean density maximum 2	%rsd	Mean density maximum 1	%rsd
Control	1.66E-06	10.2	1.35E-06	20.4		
95.5 to 96 hpf	1.33E-06	12.0	1.40E-06	12.7		
95 to 96 hpf	1.87E-06	10.8	1.42E-06	18.9	6.60E-05	30.1
94 to 96 hpf	1.31E-06	9.92	1.63E-06	12.7	3.06E-05	7.86
92 to 96 hpf	1.82E-06	6.02	1.10E-06	13.7	3.84E-05	9.40
86 to 96 hpf	1.47E-06	12.5	1.45E-06	13.1	1.44E-05	20.5
72 to 96 hpf	2.15E-06	4.69	1.28E-06	13.8	1.93E-05	22.3

References

1. Berthold MR, Cebron N, Dill F, Gabriel TR, Kötter T, Meinl T, Ohl P, Sieb C, Thiel K, Wiswedel B (2008) KNIME: The Konstanz Information Miner. In: Preisach C, Burkhardt H, Schmidt-Thieme L, Decker R (eds) Data Analysis, Machine Learning and Applications: Proceedings of the 31st Annual Conference of the Gesellschaft für Klassifikation e.V., Albert-Ludwigs-Universität Freiburg, March 7–9, 2007. Springer Berlin Heidelberg, Berlin, Heidelberg, pp 319–326

2. Petrus JA, Chew DM, Leybourne MI, Kamber BS (2017) A new approach to laser-ablation inductively-coupled-plasma mass-spectrometry (LA-ICP-MS) using the flexible map interrogation tool 'Monocle.' Chem Geol 463:76–93 . doi: 10.1016/j.chemgeo.2017.04.027
